# Computer automation for physics chart check should be adopted in clinic to replace manual chart checking for radiotherapy

**DOI:** 10.1002/acm2.13177

**Published:** 2021-02-02

**Authors:** Edward L. Clouser, Quan Chen, Yi Rong

**Affiliations:** ^1^ Radiation Oncology Department Mayo Clinic Arizona Phoenix AZ USA; ^2^ Radiation Medicine Department University of Kentucky Lexington KY USA

**Keywords:** accuracy and efficiency, computer automation, physics plan check and chart review

## 1 INTRODUCTION

In 1994, American Association of Physicists in Medicine (AAPM) task group (TG) report 40 established that plan check and chart review is part of medical physics major responsibilities.^1^ As the treatment technique complexity increases, patients’ plan check and chart review becomes more critical to treatment accuracy and patient safety, yet more cumbersome as the checking items increase dramatically. AAPM published two scientific reports in 2020 specifically to address the efficiency strategies and minimum requirements.^2^, ^3^ Both reports discussed the benefits of computer automation in reducing human labor and improving process efficiency, whereas they also emphasized the difficulty and limitation of implementing computer‐aided programs for various clinical practices. There poses a dilemma that computer‐automation can save clinical physicists’ time, while implementing a computer‐aided chart check program requires high standardization in nomenclature and continuous maintenance to accommodate ever‐changing technology and various clinical workflows. This article debates on the proposition “Computer automation for physics chart check should be adopted in clinic to replace manual chart checking for radiotherapy.” Herein, we have Mr. Edward Clouser argues for the proposition whereas Dr. Quan Chen argues against the proposition.

Mr. Clouser received M.S. in Physics in 2003 from Cleveland State University. He received his clinical training as a medical physicist at the Cleveland Clinic and stayed on faculty post‐graduation. He has worked at the Mayo Clinic in Arizona for 14 yr, among which he has been serving as the program director of the Medical Physics Residency for 7 yr. His current interests include developing tools to automate clinical work including chart review and weekly checks for dosimetrists and physicists. He holds the rank of Assistant Professor of Radiation Oncology in the Mayo School of Medicine and is board certified by the American Board of Radiology.

Dr. Chen received his PhD in Medical Physics from the University of Wisconsin‐Madison in 2004. He started his career in industry as a senior research physicist at Tomotherapy before joining University of Virginia in 2011. Currently he is an Associate Professor at University of Kentucky. His research interests cover a wide range of topics include dose calculation algorithms, motion management, adaptive therapy, kV dosimetry, innovative quality assurance method, as well as Artificial Intelligence (AI). He has cofounded a company (Carina Medical LLC) to develop AI‐based applications for Radiation Oncology. Dr. Chen has also developed many clinical tools that was in use at different centers to improve the safety and efficiency of clinical services. Dr. Chen is an Associate Editor of journal of applied clinical medical physics (JACMP) and serves on several committees at AAPM.

## 2 OPENING STATEMENT

### 2.1 Edward L. Clouser

Chart checking has long been a primary task of clinical medical physicists in the process of ensuring treatment planning integrity. Historically, we would look through a paper chart and maybe a few printed pages from the treatment planning system to verify adherence to general planning rules and finding transcription errors. The concepts of a chart check are held in the individual physicist’s head and the effectiveness in identifying errors are mostly based on individual physicist’s experience and attention to details.

As the radiation oncology treatment planning and delivery technologies evolve to a rather high level of complexity, chart checking requires a far more complicated and organized venture. Thanks to digital imaging and communications in medicine (DICOM) file standardization, record and verify systems, and other software advances, patients’ detailed treatment data can be created, transferred, and delivered in a rather secure and integrated manner. Manual transcription errors for plan and machine settings should be nearly extinct. In a single vendor environment for oncology information system (OIS), treatment planning system (TPS), and treatment delivery system, any sort of errors pertaining files transfer are eliminated.

Meanwhile, Medical Physics as an industry has moved away from “in my head” QA steps and is promoting more advanced techniques such as using checklists and other industry‐born systems like Failure Mode Effects Analysis (FMEA) and process control. AAPM TG‐100 and Medical Physics Practice Guidelines (MPPG) 4a point the direction the field is heading to.^4^, ^5^ Automation also clearly fits in “MedPhys 3.0” under the second initiative “Smart Tools.”

The AAPM TG 275 described importance of the chart check in physics QA process.^2^ The task group included a review of publications related to automation and automation tools, and listed “Develop automated tools to assist with physics plan and chart review tasks” in their “Key Recommendations” to software vendors section and also recommended “automating checks where possible” in the conclusion. TG‐275 supplement 1 included a total of 171 potential QA items for initial chart check with 109 of them as partially or fully being automated. Ending manual transcription is listed as a “Key Recommendation” in TG‐275. My proposition herein is that automation in chart checking benefits all clinical physicists and fall under the umbrella of safety. The following paragraphs will spell out how and why each benefit leads us to safety, and will also address efficiency, human error and effects of fatigue, and improvements in workflow.

The efficiency through automation is obvious: a computer can do certain tasks much faster than humans. However, there are far less obvious gains in efficiency when automating a chart check. Historically, a dosimetrist or physicist creates a treatment plan, a physician reviews it, a dosimetrist finalizes the plan, and then a physicist performs the chart check. This manual workflow is fine, so long as the physicist doesn’t find any problems. If the plan needs adjustment, there is inefficiency in the process. There may also be some awkwardness of telling someone you don’t agree with their work. In addition, introducing human to human communication with potentially emotional or subjective interactions in a workflow might add to unpredictable problems. By automating some of the plan checks and shifting the automation to occur during the planning process, the time sink of the iterative process of passing the plan between planner and checker could be avoided. Smooth, well‐defined workflows are safer workflows. This concept of automating and moving the QA to before the chart check is supported in TG‐275 as best practice.

Automation eliminates the natural burnout for human beings. If charts come in to be checked at an even pace with a predictable distribution of errors, physicists may be able to handle them with full attention. The reality is that urgent charts come in unexpectedly and sometimes multiple come in together. Clinical physicists must all have experienced the chaos that urgent patient starts in 45 min and requires immediate chart checking, while some might come in late on a Friday afternoon after a whole day of high‐intensity procedures, i.e. brachytherapy. Human nature dictates the fact that we cannot always perform at our best. On the contrary, a computer doesn’t get tired, need to eat a meal, or care if it is a Friday night. Errors can slip past our best intentions; they are far less likely to slip past a well‐written algorithm. Not letting errors get past our safety barriers is clearly a safer condition. Gopan et al. concluded in their 2016 article regarding errors not being caught that “Suggestions for improvement include the automation of specific physics checks performed during the pretreatment physics plan review and the standardization of the review process.”^6^

Automation saves time in the overall workflow, thus allowing more time be allocated to those more important checks, or steps that might be scored the highest risk in making errors in FMEA. Manual steps tend to be bottlenecks in a clinical process. The efficiency argument I started with has benefits beyond the actual chart check. Fully or partially automating any step in a process allows the workflow to move along to the next step in the process faster by removing barriers from human delays.

Automation also lends itself to meaningful data collection. If the results of every chart check are reliably collected, they can then be reviewed and analyzed. Data can be collected manually as well, I won’t deny that, but it becomes time consuming and prone to errors if it is not automated. In an institution that has multiple staff members involved in planning and chart checking, this data can be valuable in establishing patterns in practice and potentially leading to targeted practice improvement projects. All physicists can understand the power of data, and tackling any problem is much easier with data.

### 2.2 Quan Chen, PhD

Plan/Chart checking is a key step to ensure the quality and safety of radiation therapy treatment. A large‐scale study on 4407 incidents reported at 2 academic radiation oncology clinics revealed that physics initial chart review and physics weekly chart review are the two most effective quality control (QC) processes for detecting those reported high severity incidents.^7^ Chart checking is specified by AAPM^1^ and ACR‐ASTRO^8^ as an important duty for medical physicists. The recently published AAPM TG‐275 has also made recommendations for physics initial plan and weekly chart review to strengthen the effectiveness of these activities in ensuring the safety and quality of care for patients receiving radiation treatments.^2^

The advancement in technologies has tremendously increased the complexity of radiation therapy treatment. This has increased the burden for physicists to perform a thorough chart check. There have been many efforts to develop automated chart checking tools to reduce human efforts and errors. Researchers at University of Iowa have developed an electronic radiation therapy plan quality assurance (QA) system (EQS)^9^ which later becomes CATERS (Computer Aided Treatment Event Recognition System).^10^ This system checks the consistency of the plan parameters designed in the TPS compared to those in the OIS, to ensure plan transfer integrity. In addition, various logic consistency checks are implemented to alert inconsistent findings or possible errors such as target dose deviation from physician’s prescription, inappropriate parameters that are known to cause interlocks, etc. A similar system has been developed at Washington University in St. Louis before 2012.^11^, ^12^ It was subsequently expanded to include more functions such as the verification of treatment delivery through the EPID,^13^ adaptive radiotherapy,^14^ proton therapy,^15^ and MR guided radiotherapy.^16^ Researchers at University of Michigan (UM) developed a Plan‐Checker Tool (PCT) to automate part of the chart checking tasks.^17^ Commercial vendors have also released a few solutions to facilitate plan/chart checking tasks, including ClearCheck/ChartCheck from Radformation Inc., Mobius3D/MobiusFX from Varian Medical Systems, and PlanCheck/PlanIQ from Sun Nuclear Corp. The plan/chart checking functions provided by these vendors are similar to those in‐house developed at academic centers.

Although automated chart checking tools, both in‐house and commercially developed, are available, none of them are even close to fully replacing manual checks. The automated checking functions offered are only a very small subset of the actual checks performed by physicists. For example, the PCT system which was developed fairly recently (2016) only automated 19 of 33 checklist items identified at their institution. Note that the recently published AAPM TG‐275^2^ Table S1.A.ii listed over 170 physics check items for photon/electron EBRT initial plan/chart review and Table S1.A.iii showed that 87 of them have failure modes of RPN > 100. So far, none of the software claimed to be able to fully replace manual checks or reviews.

There are many obstacles preventing the implementation of an automated system that can replace physicists in plan/chart checking. The automated chart checking functions implemented so far mostly rely on the entry and existence of structured data. A number appeared in one data field will be compared with a number appeared in the other data field or a box checked somewhere. However, the data in the patient chart are not always structured. There can be key information entered as a free text in the form of a note. Often, it can simply exist in the patient chart as a scanned document (i.e. patient’s prior treatment record is often faxed from a different clinic). While it is easy for human to understand the information carried in those texts, computer apprehension requires optical character recognition (OCR) and natural language processing (NLP) that confound computer scientist for over 50 yr. While only recently, the success of IBM Watson in Jeopardy! showed promise in this area, the subsequent failures of IBM’s attempt to adopt it in the medical field showed discouraging obstacles.^18^ Similarly, an important aspect during plan/chart checks involves image review, that is, to evaluate contours accuracy or appropriate image fusion. While there are research attempts to perform contour quality assurance with computer algorithms,^19^, ^20^ no literature has shown the automation of image review for plan/chart checks.

While it is foreseeable that the advancement of computer technologies, especially the artificial intelligence technologies, might allow us to implement computer automation in every chart checking task, a remaining obstacle for creating an automated chart checking software is to handle the ever‐evolving technology development and ever‐changing patient’s individual scenarios in radiation oncology practice. Currently on the market exists numerous combinations of treatment modalities, treatment planning systems, OISs, etc. To be able to handle all systems requires tremendous knowledge and efforts. All in‐house developed solutions only focus on specific configuration for the developer’s institution. Even for commercial software, the support of different systems can be limited. For example, the ClearCheck/ChartCheck from Radformation Inc. only supports Eclipse (Varian Medical System). Furthermore, the clinical practice also varies between institutions and between physicians in the same institution. There can also be changes to clinical practices as new recommendations on treatment emerge, which further limits the general utilization of an automated chart checking system developed for one particular institution and creates maintenance issues when changes occur in clinical practice, that is, roster changes or new technology adoption. For example, some of the automation of chart checking tasks require certain naming convention,^17^ a different clinic adopting this automation would involve either changing their naming convention, or modifications in the automation software. Therefore, the high maintenance of such software in a highly variable and rapidly changing environment might not necessarily lead to a labor or time saving.

As with any software program, automated chart check can have “bugs”. Aside from programmer’s mistakes, the most common “bugs” in the program often originate from the design of the chart checking program. Usually, the chart check logics (checklists) used in manual chart check is implemented. Known errors captured with manual chart checks in the past can be used to test the program. There is a major logical flaw in this design, that is, *you cannot catch an error that you did not foresee*. Rarely occurred errors may not be considered during the implantation of automated chart checking programs. However, rarely occurred errors can still cause severe outcomes. There have been reports on errors missed by the automatic chart checking program.^9^ Although “patches” are normally developed to address these errors, they cannot address other unforeseeable errors, which might require endless program patches, thus exhausts implementing physicists or IT technicians. Therefore, completely relying on the automated QA can be impractical or even dangerous.

Finally, automated chart checking programs can only analyze information documented in charts. However, if the error occurs at the documentation step, it may not be caught by analyzing the chart itself. Often, these errors might come with high severity. For example, the “Miscommunication about prior dose, pacemaker, or pregnancy” has the 2nd highest RPN score among photon/electron EBRT high‐risk failure modes according to TG‐275.^2^ If the prior treatment checkbox in the patient chart was accidently left unchecked (although the medical resident in charge of this patient knows about the prior treatment and requested the prior treatment dose), the chart checking program will still believe that the patient has no prior treatment and performs routine chart check accordingly. However, a physicist checking this case may capture the prior treatment information of the patient from various venues, i.e. chart rounds, dosimetry huddle, emails communications, or additional external dicom files for this patient. Human wisdom, experience, and communication abilities can never be replaced by rule‐following robots.

## 3 REBUTTAL

### 3.1 Edward L. Clouser

I would like to start my rebuttal by saying I agree with nearly everything my opponent has laid out. I don’t think we can replace people with automation, today. I do think that we can and should find as many things as possible to automate with full automation as a goal, not an ultimatum.

We should look at chart checking automation as a spectrum, not a Boolean. Most technologies evolve, and most are very “ho‐hum” or even dangerous when they’re new. I can get on a plane from my home in Phoenix and be in London, 5300 miles away, in less than half a day. If we took the plane the Wright brothers flew and determined it was dangerous and therefore not worth pursuing, that journey would take weeks, not hours. Even today, planes are not 100% safe, but we all accept a small amount of risk for the massive rewards. I would never trivialize the loss of life or minimize the importance of what we do as Medical Physicists. In fact, I’m trying to make the opposite argument, that the human can’t be trusted to achievement improvement on their own for the very important quality assurance duties we perform. We need to commit to automation in order to aid the evolution and to keep it as safe as possible. Just like human flight, the end result will be worth the potential problems along the way.

Most arguments to avoid automated chart checking fall in a classic human emotional bias known as “status quo bias.” The current state of affairs is viewed as a reference point and any move from it, (regardless of direction!) is perceived as a loss. This was well described in the results of experiments by Samuelson and Zeckhauser in their 1988 article in the Journal of Risk and Uncertainty.^21^ In summary, when given a choice, humans will more likely pick what they have, rather than something else, even if the alternative has clear benefits. Minor examples in our everyday lives might be keeping our current insurance company or mobile phone carrier, even though switching could save us money. Everyone’s bias level is different, but we tend to keep what we have.

My opponent’s last argument for human vs. automated chart checking is that a human might have better information in making a decision; perhaps because they attended Chart Rounds or read something outside of the Record and Verify system. I agree that a state with more data is a better state than less. That just means that data needs to get to the automation, not an abandonment of the data. Human’s miss errors all the time and we collectively learn from those errors. The entire purpose of programs like AAPM/ASTRO’s ROILS (Radiation Oncology Incident Learning System) is to learn from mistakes. Adding or altering code is no different than learning about an incident and adjusting your practice to prevent that mistake at your institution. The biggest difference being the code won’t forget over time, you and I might.

### 3.2 Quan Chen, PhD

“Awkwardness of telling someone you don’t agree with their work,” “human to human communication … might add to unpredictable problems.” My opponent considered human to human communication negative, which should be avoided if possible. However, I believe in‐person communication is the major advantage of having human touches vs. using machine/automated tools. Communication includes two vital aspects: express yourself and understand others. As mentioned in my opening statement, clinic is a complex and dynamic environment. Errors can happen due to various reasons. In addition, false‐positives could be generated from a chart checking routine that did not fully consider some of the peculiar or rare cases. Human to human communication renders quick and comprehensive understanding of the circumstances, possible sources of errors or false‐positives, and solutions that reduce or even prevent future errors. All the above can lead to amendment of our chart check routines, in order to better eliminate unconventional sources of errors in rare scenarios. Physicist should not be afraid or feel awkward of speaking out on the matter of patient safety.

There is no question that certain chart checking tasks could and should be automated. The simple comparison of well‐structured data between TPS and the OIS is such an example. However, as detailed in my opening statement, the complex nature of our practice environment will lead to complex rules in the chart checking algorithm. As the complexity of the system grows, so does the possibility of errors and the difficulty to fully validate it. In addition, there are many unstructured data, images, and information outside of the chart that is difficult to be handled by the automated chart checking algorithms. The most dangerous aspect of (mis‐)using the automated chart checking tool is that user may not fully understand the rules and limitations. There can be false or misleading advertisement of a chart checking tool that it can catch certain error without mentioning the fact that it might only check one error in the workflow among many that could lead to a specific error.

A full automated tool that can cover all aspects of chart checking, even if it can be built, will usually only cover the existing clinical scenarios (machine capabilities, treatment schemes, clinical workflows, report formats, etc.). As clinical practice keeps evolving, the previously “perfect” tool may fail to cover all the bases. It is then up to the human physicist to ensure the safety of the treatment, which includes a thorough chart checking, before new “patches” can be developed. However, it is very likely that the physicists may already have been rusty on chart checking skills as they have been relying on the automated chart checking tool for too long.

We believe that while the automation of chart checking is beneficial, it will not and should not fully replace manual chart checking. The focus of the effort, should not be on the development of a complete system that can automate chart checking under any clinical environment and able to capture all possible errors. Instead, the effort should be on the development of a set of tools that can perform some, well defined chart checking tasks. Physicists should have a full understanding of the function, logic, and limitations of these tools. However, it should still be human physicists who will consolidate the information provided by these automated tools, as well as other information inside and outside of the charts, to determine whether a treatment can be safely administrated.
